# Diagnostic Workup for Patients with Solid Renal Masses: A Cost-Effectiveness Analysis

**DOI:** 10.3390/cancers14092235

**Published:** 2022-04-29

**Authors:** Jasmin Runtemund, Johannes Rübenthaler, Niklas von Münchhausen, Maria Ingenerf, Freba Grawe, Gloria Biechele, Felix Gerhard Gassert, Fabian Tollens, Johann Rink, Sasa Cecatka, Christine Schmid-Tannwald, Matthias F. Froelich, Dirk-André Clevert, Moritz L. Schnitzer

**Affiliations:** 1 Department of Radiology, University Hospital, LMU Munich, Marchioninistr. 15, 81377 Munich, Germany; jasmin.runtemund@med.uni-muenchen.de (J.R.); maria.ingenerf@med.uni-muenchen.de (M.I.); freba.grawe@med.uni-muenchen.de (F.G.); gloria.biechele@med.uni-muenchen.de (G.B.); sasa.cecatka@med.uni-muenchen.de (S.C.); christine.schmid-tannwald@med.uni-muenchen.de (C.S.-T.); dirk.clevert@med.uni-muenchen.de (D.-A.C.); moritz.schnitzer14@gmail.com (M.L.S.); 2 Department of Radiology and Nuclear Medicine, University Medical Centre Mannheim, Theodor-Kutzer-Ufer 1-3, 68167 Mannheim, Germany; niklasvon.muenchhausen@umm.de (N.v.M.); fabian.tollens@umm.de (F.T.); johann.rink@umm.de (J.R.); matthias.froelich@umm.de (M.F.F.); 3 Department of Diagnostic and Interventional Radiology, Klinikum rechts der Isar, Technical University of Munich, Ismaninger Str. 22, 81675 Munich, Germany; felix.gassert@tum.de

**Keywords:** CEUS, cost-effectiveness, solid renal masses

## Abstract

**Simple Summary:**

There are several benign and malignant types of solid renal masses. For diagnostic and characterization of these masses, a few imaging methods such as magnetic resonance imaging (MRI), computed tomography (CT) or (contrast-enhanced) ultrasound (CEUS) are established in the clinical routine. The aim of our study was to assess the most economical approach for detecting and characterizing these masses. As a result, contrast-enhanced ultrasound turned out to be a cost-effective diagnostic method. Therefore, if available, this method should be considered in the routine. Alternatively, MRI also offers excellent diagnostic accuracy, but it is associated with higher costs. This result may lead to a change in the diagnostic workup of solid renal masses in clinical routine, as contrast-enhanced ultrasound should be considered as an appropriate method for the first analysis compared to CT and MRI.

**Abstract:**

Background: For patients with solid renal masses, a precise differentiation between malignant and benign tumors is crucial for forward treatment management. Even though MRI and CT are often deemed as the gold standard in the diagnosis of solid renal masses, CEUS may also offer very high sensitivity in detection. The aim of this study therefore was to evaluate the effectiveness of CEUS from an economical point of view. Methods: A decision-making model based on a Markov model assessed expenses and utilities (in QALYs) associated with CEUS, MRI and CT. The utilized parameters were acquired from published research. Further, a Monte Carlo simulation-based deterministic sensitivity analysis of utilized variables with 30,000 repetitions was executed. The willingness-to-pay (WTP) is at USD 100,000/QALY. Results: In the baseline, CT caused overall expenses of USD 10,285.58 and an efficacy of 11.95 QALYs, whereas MRI caused overall expenses of USD 7407.70 and an efficacy of 12.25. Further, CEUS caused overall expenses of USD 5539.78, with an efficacy of 12.44. Consequently, CT and MRI were dominated by CEUS, and CEUS remained cost-effective in the sensitivity analyses. Conclusions: CEUS should be considered as a cost-effective imaging strategy for the initial diagnostic workup and assessment of solid renal masses compared to CT and MRI.

## 1. Introduction

In modern medicine, imaging is one of the most important strategies in medical diagnostics. Due to technical progress, several different modalities are available for physicians to provide the best care for their patients. As the number of examinations increases, the number of incidentally found diseases also increases. A typical example of this matter is the incidental detection of solid renal masses in 13–27% of adults undergoing cross-sectional abdominal imaging [1]. Solid renal masses are divided into benign and malignant neoplasms. Benign neoplasms of the kidney are angiomyolipoma and oncocytoma. The malignant tumor of the kidney is renal cell carcinoma (RCC). RCC is the sixth most diagnosed cancer in men and the tenth most diagnosed cancer in women. with 140,000 deaths worldwide per year, which makes RCC one of the most lethal neoplastic diseases [2,3]. Hypertension, obesity, smoking and chronic kidney disease are the most important risk factors for RCC [2]. The peak age is between 60 and 70 years [4]. RCC can be classified into different subtypes. The most common subtype is clear cell carcinoma, with 65–80% of renal cell carcinomas [5,6]. Additionally, 10–15% of RCCs are papillary carcinomas, and 4–11% are chromophobe carcinomas [3,7,8]. The standard of treatment for RCCs is partial nephrectomy, radical nephrectomy and active surveillance [4]. In recent years, several minimally invasive treatment options such as ablation or a laparoscopic approach have become more important in treating RCCs, as they are an alternative for patients who are not suited for surgical treatment [9,10,11,12]. The recently published amendments to the guidelines of the American Urological Association (AUA) for the management of solid renal masses recommend the use of thermal ablation as a preferred technique in cT1a patients to reduce mortality. Further, Bosniak 3 and 4 complex cystic renal masses should be treated with radical nephrectomy if there is an increased oncological risk [13,14]. The majority of diagnosed RCCs happen to be incidental during routine abdominal imaging with ultrasound (US) or computed tomography (CT); therefore, the incidence of RCCs has increased significantly in recent years, as the amount of cross-sectional diagnostics has grown [1,2,4,10,15]. The imaging technique of choice is CT, as it offers high spatial resolution, fast examination, widespread availability and relatively low costs [9,15,16,17]. However, there are several pitfalls of CT in differentiation between malignant and benign tumors, as 20% of masses with a size <4 cm are benign oncocytomas or angiomyolipoma that cannot be certainly differentiated. Furthermore, CT bears the risk of contrast-induced nephropathy and contains a non-negligible radiation dose [15,18]. Thus, for further characterization of such masses, magnetic resonance imaging (MRI) is better suited, as it offers a broad spectrum of advantages. MRI offers superior soft-tissue contrast and functional imaging, does not contain ionizing radiation and allows the characterization of lesion vascularity and diffusion restriction. This enables the physician to differentiate between benignancy and malignancy even in their histological grade [3,4,5,9,16]. Therefore, imaging not only goes beyond diagnosis itself, but also takes a key part in ensuring suitable patient management in order to avoid unnecessary interventions [3]. On the other hand, MRI diagnostics are rather expensive and are very time-consuming [9,16]. Besides the fact that CT and MRI have their advantages and disadvantages and complement each other, this leaves a diagnostic gap between these two imaging modalities. In recent years, contrast-enhanced ultrasound (CEUS) has emerged as a backup modality that could close the gap between MRI and CT. Until now, CEUS has only been considered as a complementary option to CT and MRI in RCC diagnostics [17,19]. Nonetheless, CEUS provides considerable advantages, as it is non-invasive, rather cheap, widely available and is independent of thyroid or renal function [1,17,18,20,21,22]. So far, CEUS has shown excellent diagnostic performance in renal imaging [23,24]. Nevertheless, in modern medicine, not only the diagnostic performance, but also the economical point plays a significant role in choosing an adequate strategy [25,26,27]. It must be emphasized that not only should the initial costs of diagnostics be decisive, but also the long-term costs should be considered more in detail, as a method may seem more expensive in the first place, but in the long term, a false-negative diagnostic would lead to significantly higher treatment costs and would worsen patients’ health states. Therefore, several imaging methods must not only be evaluated by their diagnostic performance, but also by their cost-effectiveness. Yet, the economic efficiency of CEUS compared to CT and MRI in the imaging of solid renal masses is still questioned.

Consequently, this study analyzes the cost-effectiveness of CEUS, CT and MRI in the diagnostic imaging of solid renal masses.

## 2. Results

### 2.1. Economic Analysis

#### 2.1.1. Estimated Outcomes and Related Expenses

The findings of our analysis were assessed in a Markov model. Therefore, patients without solid renal masses and masses without therapy, patients with solid renal masses and in time therapy and patients with solid renal masses and a delayed therapy were modeled similarly. Patients with solid renal masses had projected initial expenses of USD 4231.00, followed by monthly expenses of USD 2148.25 in the first year and USD 108.50 in the following years, with an additive quality of life (QoL) of 0.75 QALYs. In contrast, patients with delayed therapy led to initial expenses of USD 6346.50, monthly expenses of USD 2086.42 in the first year and USD 810.58 in the following years, with an additive QoL of 0.66 QALYs. Additionally, healthy patients with an initially indicated treatment turned out to have an additive QoL of 1 and an additive expense of USD 1302.00 and USD 1375.00 for TN and FP, respectively.

#### 2.1.2. Cost-Effectiveness Analysis

**Figure 1:**
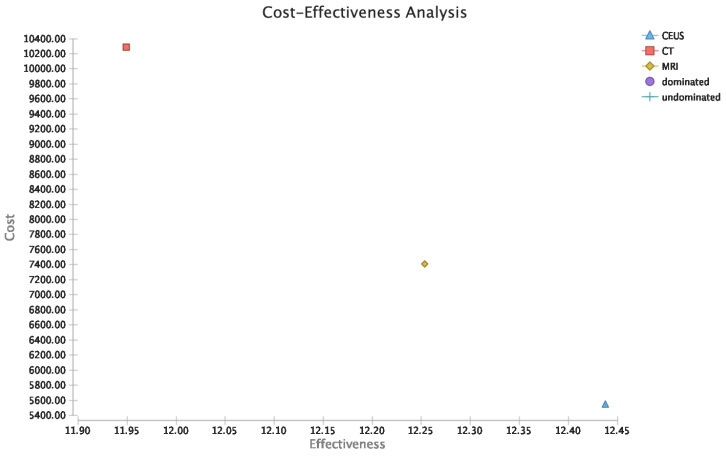
Cost-effectiveness analysis.

In view of the outcomes of the model calculations, the cost-effectiveness analysis in the reference case scenario CT led to an efficacy of 11.95 QALYs and total expenses of USD 10,285.58. MRI led to an efficacy of 12.25 and total expenses of USD 7407.70, whereas CEUS led to an efficacy of 12.44 and total expenses of USD 5539.78 (Figure 1). Accordingly, the equivalent incremental cost-effectiveness ratio (ICER) of MRI was −10,115.05 and 9703.86 for CT.

#### 2.1.3. Deterministic Sensitivity Analysis

**Figure 2:**
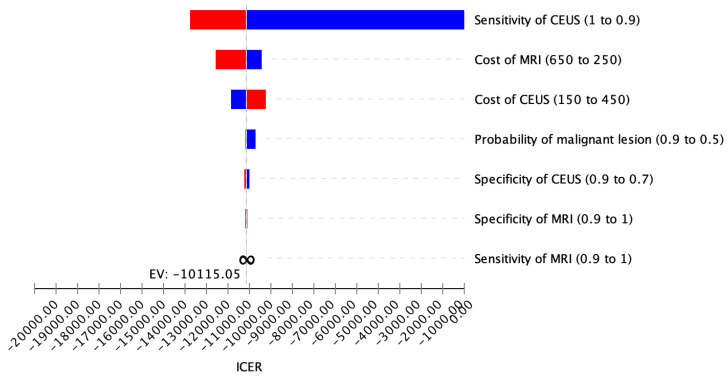
Deterministic sensitivity analysis. The displayed tornado diagram demonstrates the influence of changing values on ICER in the reference case. The ICER of CEUS remained beneath the WTP boundary of USD 100,000 per QALY for every analyzed parameter range, proving the cost-effectiveness of CEUS in the base-case setting.

For the investigation of the strength of the model, a deterministic sensitivity analysis containing expenses and diagnostic efficacies, in particular specificities and sensitivities of CEUS, CT and MRI, was carried out. CEUS turned out to result in an ICER beneath the willingness-to-pay (WTP) boundary of USD 100,000 per QALY in the foreseen ranges, pointing out the economic superiority of CEUS (Figure 2).

#### 2.1.4. Probabilistic Sensitivity Analysis

**Figure 3:**
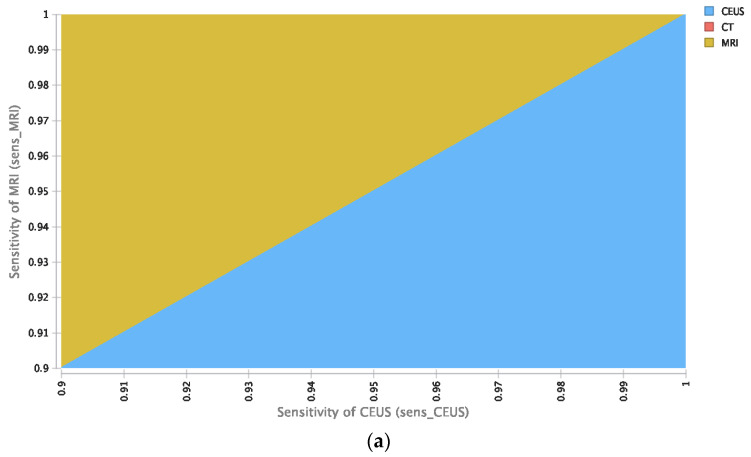

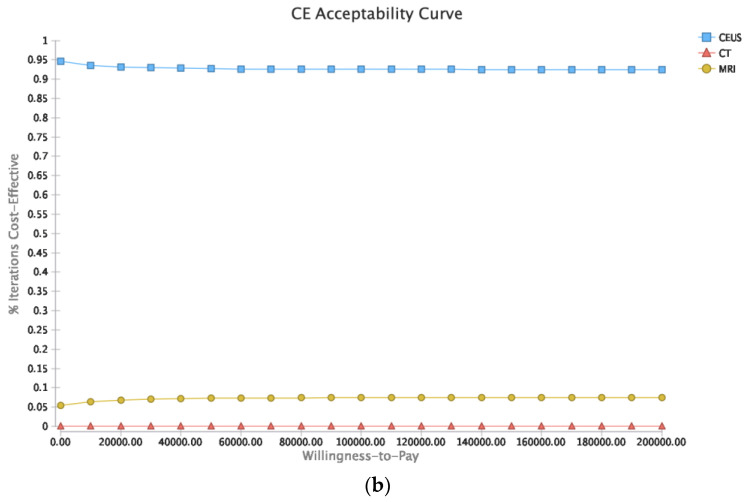
Probabilistic sensitivity analysis. (**a**) Various sensitivity boundaries of CEUS and MRI and their impact on cost-effectiveness at a WTP of USD 100,000/QALY. (**b**) Acceptability of diagnostic strategies out of 30,000 reiterations for different WTP boundaries indicating CEUS as the most economical approach in detection and characterization of solid renal masses.

For additional analysis of the stability of our calculations, a probabilistic sensitivity analysis on the basis of the utilized input parameters explained in Table 1 was executed (Figure 3a). At the elected WTP, CEUS was economic in the majority of repetitions (Figure 3b).

## 3. Discussion

This study exhibits that CEUS is a cost-effective imaging strategy in the diagnostic workup and differentiation of solid renal masses compared to CT and MRI. These results differ from the current guidelines for renal mass imaging recommending MRI and CE-CT as the gold standard and utilizing CEUS only as a complementary option in special cases [17,19].

The use of CEUS or MRI for the identification and characterization of solid renal masses is superior to CT, as they both show comparable high sensitivities and are very cost-saving, as the therapy streamlining is superior. CEUS additionally has the advantage of a significantly lower cost point; therefore, the streamlining is even stronger. As a matter of fact, treatment planning and surgical management of malignant and benign solid renal masses require a very precise differentiation between different RCC subtypes or between malignant and benign renal masses. This differentiation issue especially concerns the distinction between RCC, oncocytomas and fat-poor AMLs with MRI and CT. Even though MRI is known to have superior soft-tissue contrast, MRI has its struggles in the detection of fat-poor AMLs. These fat-poor AMLs are a subtype that accounts for 5% of AMLs and are defined by having only a small amount of fat compared to classical AMLs with a substantial amount of fat [4]. At T2WI, fat-poor AML seems to be hypointense and therefore present similar to papillary RCCs. This uncertainty in diagnosis often requires an additional biopsy to assure accurate diagnosis [16]. Not only fat-poor AMLs, but also oncocytomas are very challenging to distinguish from RCC in MRI and CT [19,35,36]. Although only 2–3% of non-fat-containing renal tumors are oncocytomas, diagnostic differentiation is key to avoiding unnecessary biopsy [36].

As a matter of fact, the cost-effectiveness of CEUS in the diagnostic workup of solid renal masses is not an exception. There are currently several studies that have investigated the cost-effectiveness of CEUS in variable types of lesions and tumor entities. A study in 2020 showed that CEUS is the most economical approach for the evaluation of unclear cystic renal lesions compared to MRI [37]. Moreover, a study in 2019 questioned the cost-effectiveness of CEUS for the characterization of non-palpable testicular lesions [38]. In a similar manner, CEUS turned out to be the cost-effective modality compared to color Doppler ultrasound and native B-mode [38]. Further, in 2013, Westwood et al. investigated the cost-effectiveness in the characterization of focal liver lesions and detection of liver metastases. In this study, CEUS is compared with contrast-enhanced CT (CE-CT) and contrast-enhanced MRI (CE-MRI). The diagnostic performance for detecting and characterizing focal liver lesions of CEUS was comparable to CE-CT and CE-MRI; nonetheless, the cost-effectiveness analysis proved that CEUS is an economical alternative to CE-MRI and CE-CT and should be rated as cost-effective [39]. This study is in line with our investigation, as it shows that CEUS is not only superior in renal imaging, but is also a considerable method in liver tumor diagnostics.

**Figure 4:**
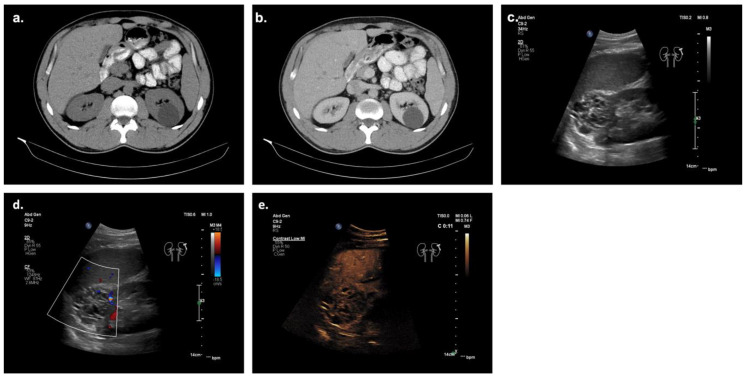
Patient from our institution undergoing diagnostic workup of clear cell RCC. (**a**) Native CT showing a hypodense lesion in the left kidney without any signs of solid components. (**b**) CT in venous phase showing a hypodense lesion in the left kidney without contrast-uptake or nodular components. (**c**) B-mode ultrasound of the left kidney shows a partly hypo- and partly hyperechoic lesion that does not fulfill the sonomorphological criteria for a cystic lesion. (**d**) Color doppler ultrasound of the lesion shows major vascularization inside the solid lesion. (**e**) CEUS of the solid lesion proves major vascularization of the solid lesion in the arterial phase with partly necrotic parts, which do not appear vascularized, in line with sonomorphological features of a malignant renal tumor. The diagnosis of clear cell RCC was confirmed histologically after surgical excision.

In Figure 4, the diagnostic workup of a case of our institute with suspicious solid renal mass is shown in native CT and in venous phase, B-mode and CEUS. The initial diagnostic choice was CT showing a hypodense area in the left kidney in the native and venous phases. However, a further assignment between RCC or benign lesions with only CT is hardly possible. For further evaluation, native B-mode ultrasound and CEUS were performed, proving the superiority of diagnostic value for renal mass evaluation, as it could clearly be characterized as an RCC. Most likely, the same outcome could have been achieved with an MRI examination. Still, the examination costs and duration of CEUS are significantly lower than MRI, indicating CEUS as the modality of choice for the initial examination.

In general terms, political and structural changes in health care systems cannot solely be based on medical evidence, as they require an economic rationale in terms of cost-effectiveness regarding distributive justice. Therefore, cost-effectiveness analyses, as performed in the current study, are an important tool to provide a more comprehensive data basis to decision makers in health care and politics than clinical trials alone [40]. However, indeed, there are occasional worries among healthcare professionals about the effectiveness of clinical decision making and about the benefit of single economic metrics to address this issue [41]. For instance, it is questioned whether the incremental cost-effectiveness ratio (ICER) as a parameter for clinical decision making will actually reduce non-efficient therapies and optimize the patients’ medical attention. It needs to be outlined that cost-effectiveness analyses serve the healthcare system not by unreserved improvement of every patient’s treatment without regarding the individual circumstances, but by giving the healthcare providers a guide to value each treatment option by its benefit for the patient and the healthcare system. As a matter of fact, improved diagnostics due to high diagnostic accuracy yield the problem of overdiagnosis, especially in patients without any clinical signs or symptoms that would have led to treatment in the first place. However, we strongly believe that the evaluation of the cost-effectiveness of the diagnostic modalities could lead to a shift in imaging towards the best-suited modality, even though the initial examination costs are higher, as the cost-effectiveness is valued for longstanding outcomes. This may have a significant impact on the patients’ long-term outcomes and could be beneficial for the patients’ quality of life. Furthermore, willingness-to-pay (WTP) should be similarly regarded as just one of many indicators that may be of interest for comprehensive economic consideration. Commonly used WTP boundaries between USD 50,000 and USD 100,000 per quality-adjusted life year (QALY) should not be considered as absolute borders for every treatment, as every patient is an individual, and so is their treatment.

Although CEUS was by far the cost-effective strategy in the initial diagnostic workup of solid renal masses in the baseline scenario, some limiting factors need to be admitted and considered. Our calculations are affected by the dependence on the utilized input parameters, as they do not constantly reflect daily clinical reality. Hence, input parameters happen to deviate on a case-by-case basis. As a matter of fact, our results strongly depend on the attending physicians’ skills in CEUS imaging. As the sensitivities and specificities of the utilized analysis reflect the expertise of experienced physicians, the diagnostic outcomes may strongly vary between physicians with different skill grades. On a wider clinical landscape, most physicians with average skills will not achieve sensitivity and specificity values as high as used in the analysis, as CEUS is not that common in clinical routine. Therefore, the experience of each physician is the main influence on the diagnostic quality of a CEUS examination. Most likely, the average radiologist would have more sensitive and specific radiological findings with an MRI examination, as MRI is one of the basic skills learned in radiological training, whereas CEUS still fills a niche. However, if CEUS is seen as the gold standard in the diagnostic workup of solid renal masses, the number of CEUS examinations will rise, and consequently, the experience and the quality of diagnosis will also improve. All in all, MRI should still be considered as a great modality for imaging modality in the diagnostic workup of solid renal masses, and CEUS should not completely replace MRI, but rather should be a combination of those two imaging modalities considered the future gold standard. In this combination, CEUS should be regarded as the modality of choice for initial workup and assessment, and MRI should be used for further evaluation and treatment planning. Another limitation is the expense factor. In the health economy, the resources that are available for diagnostics are limited. These bounded resources have to be used in a cost-effective manner to guarantee the best utility for all patients in a healthcare system. As the costs for certain examinations may vary from time to time, the cost-effectiveness of those examinations could change. Consequently, if the costs of CEUS increase and those of MRI decrease heavily, CEUS may lose cost-effectiveness. Nonetheless, our outcome of proving the cost-effectiveness of CEUS could lead to a change in the diagnostic workup of solid renal masses, as it shifts the gold standard away from CT to a combinational approach of CEUS and MRI. Furthermore, our model simplifies the potential health states and breaks them down into three stages of disease and death. In clinical reality, the potential stages of an oncologic disease are far more individual, and there are a lot more subgroups. Nonetheless, as it varies in each patient, we needed to simplify these stages in order to gain a comprehensible and sound outcome.

All in all, from an economic perspective, CEUS can be seen as a cost-effective approach for the initial workup and assessment of solid renal masses.

## 4. Materials and Methods

### 4.1. Model Design

**Figure 5:**
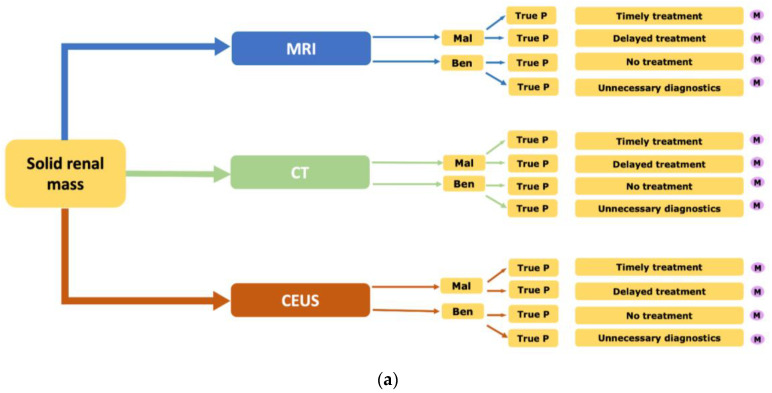

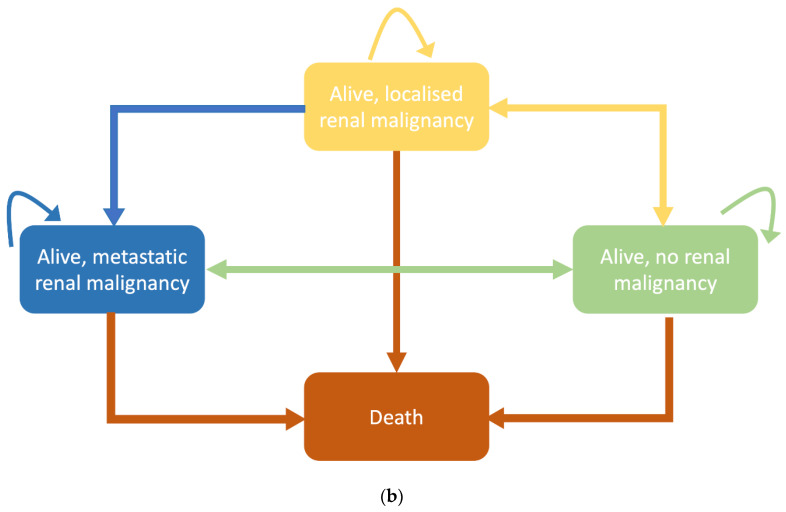
Markov layout. (**a**) A decision matrix for every strategy integrating CEUS, MRI and CT. For every result, a Markov calculation was executed. (**b**) Markov model for solid renal masses with potential stages “Alive, metastatic renal malignancy”, “Alive, localized renal malignancy”, “Alive, no renal malignancy” and “death”. The starting stage was specified dependent on the results in the decision model.

For the assessment of the cost-effectiveness of the diagnostic workup of solid renal masses, a Markov model was designed utilizing a decision–analytic software (TreeAge Pro Version 19.1.1, Williamstown, MA, USA). A decision model for every diagnostic strategy represents the decision tree of our Markov model. The strategies are the diagnostic modalities “MRI”, “CEUS” and “CT”. Every diagnostic modality can detect a malignant or benign mass with a defined sensitivity and specificity that leads to different decisions indicating certain disease management. These decisions can be “True positive”, which leads to a “Timely treatment”, “False negative”, which leads to a “Delayed treatment”, “True negative”, which indicates “No treatment” and “False positive”, which turn out to be an “Unnecessary diagnostic”. For every of these decision paths, a Markov model is performed. The decision model is displayed in Figure 5a.

A Markov model can be defined as a model for the stochastic assessment of patients’ long-lasting outcomes by valuing the assumed possibility of varying states and the possibility of transition between the different states. At each point of the model, the patient can be assigned to a health state. The Markov model contains the states “Alive, metastatic renal malignancy”, “Alive, localized renal malignancy”, “Alive, no renal malignancy” and “Death”. At the beginning of a new model cycle, in our model, every month for 1 year, the state of the patient can alter according to the predefined probabilities. Each health state can be assigned to a QoL and the assumed costs for this certain state. A graphical summarization of our model is shown in Figure 5b. The methodology of the study is based on the CHEERS checklist (Supplement Figure S1).

### 4.2. Input Values

Patients’ expected age at diagnostic procedure was 62 years as reported by previously released studies [17]. There is a 3.00% discount on costs and utilities [28]. Further, the WTP boundary was defined to be USD 100,000 per quality-adjusted life years (QALY). A general summary is displayed in Table 1. Our model can be seen from the US healthcare perspective and calculated in United State dollars (USD). US Life Tables were utilized for the assumption of age-specific risk of death.

#### 4.2.1. Diagnostic Performance

MRI sensitivity and specificity were defined to be 90% and 96%, and CT sensitivity and specificity were defined to be 75% and 72% based on comparative research work [29]. CEUS sensitivity and specificity were defined to be 99.10% and 80.50% [17].

#### 4.2.2. Expenses and Utilities

Treatment expenses of CT, MRI, CEUS, biopsy and surgery were Medicare prices in 2020. Additionally, the expenses for delayed surgery and unnecessary biopsy were incorporated into the investigation. Further, the monthly expenses of localized and metastatic tumors were obtained from the literature [30].

To measure the utilities, quality-adjusted life years (QALYs) were collected with regard to the patients’ medical condition [31].

#### 4.2.3. Transition Possibility

In consonance with the already-described decision model, possibility of initial non-R0 resection, recurrence after resection, metastatic occurrence and successful surgery of recurrence were utilized. Further, additional risk of death with localized tumor and with metastases was taken into consideration. Additionally, as an approximation of the possibility of demise not caused by a tumor burden, our calculations relate to the risk set in US Life Tables.

### 4.3. Cost-Effectiveness Analysis

Anticipated outcomes of expenses and utilities were calculated for the base-case strategy in accordance with the defined WTP and discount rate described previously. Furthermore, to assess the economic effectiveness of the diagnostic strategies, incremental cost-effectiveness ratios (ICERs) were assessed.

### 4.4. Definitions

Incremental cost-effectiveness ratio (ICER): The economic value of variating therapy strategies is plotted by the ICER. The formula for calculating the ICER is:

(1) $$ICER=\frac{\left(E_{1}-E_{0}\right)}{\left(O_{1}-O_{0}\right)}\;$$

with O_1_ and E_1_ and O_0_ and E_0_ indicating outcome and expense of each approach. The result represents the added expenses to the strategies for each QALY.

Willingness-to-pay (WTP): From a health-economical point of view, a WTP threshold refers to an assumed boundary of costs for a specific health-related benefit for the patient a healthcare system has agreed to pay.

Sensitivity analysis: An approach to assess the influence on a dependent value by altering another input value. Therefore, the result can display how uncertain a model is in its design.

Deterministic sensitivity analysis: An alteration of input values in 1-way, 2-way or 3-way sensitivity analysis.

Probabilistic sensitivity analysis: A simulation of the outcome of a model over a large number of reiterations (30,000) to figure out the possible variability of outcomes not only through the application of rigid values, but also through the consideration of the values’ range of distribution.

Acceptability curve: A pictorial method to display the percentage number of reiterations in which the analyzed modalities resulted to be cost-effective and are accepted as the most economic approach.

### 4.5. Sensitivity Analysis

For the analysis of the stability of our model prototype, probabilistic and deterministic sensitivity analyses were executed. In the case of the latter, a total of 30,000 Monte Carlo repetitions were applied. Founded on the results of the probabilistic analysis, the acceptance of a diagnostic strategy was estimated in curves.

## 5. Conclusions

In summary, CEUS should be seen as a cost-effective approach for the initial diagnostic workup and assessment of solid renal masses.

## Figures and Tables

**Table 1:** Input values.

Name	Estimate	Distribution	Source
Pre-test probability of malignant lesion	83.1%	**β**	Rübenthaler et al., 2018 [17]
Expected value at diagnostic procedure	62	**β**	Rübenthaler et al., 2018 [17]
Assumed WTP	USD 100,000.00		Sanders et al., 2016 [28]
Discount rate	3.00%		Sanders et al., 2016 [28]
Markov model time	1 year		Sanders et al., 2016 [28]
**Diagnostic test performances**			
Sensitivity of CT	75%	**β**	van Oostenbrugge et al., 2018 [29]
Specificity of CT	72%	**β**	van Oostenbrugge et al., 2018 [29]
Sensitivity of MRI	90%	**β**	van Oostenbrugge et al., 2018 [29]
Specificity of MRI	96%	**β**	van Oostenbrugge et al., 2018 [29]
Sensitivity of CEUS	99.10%	**β**	Rübenthaler et al., 2018 [17]
Specificity of CEUS	80.50%	**β**	Rübenthaler et al., 2018 [17]
**Expenses (Acute)**			
CT	USD 233.00	**γ**	Medicare (74,176)
MRI	USD 381.00	**γ**	Medicare (74,182)
CEUS	USD 285.00	**γ**	Medicare (C9744)
Biopsy	USD 1375.00	**γ**	Medicare (50,200)
In time surgery + treatment (true positive)	USD 4231.00	**γ**	Medicare (52,355)
Delayed surgery + treatment (false positive)	USD 6346.50	**γ**	Assumption (1.5×)
Unnecessary biopsy (false positive)	USD 1375.00	**γ**	Medicare (50,200)
No further action (true negative)	USD 0.00	**γ**	Assumption
**Expenses (Long Term)**			
Monthly expenses without tumor	USD 108.50	**γ**	Hollenbeak et al., 2011 [30]
Monthly expenses with detected tumor (1st year)	USD 2148.25	**γ**	Hollenbeak et al., 2011 [30]
Monthly expenses with detected tumor (after 1st year)	USD 212.67	**γ**	Hollenbeak et al., 2011 [30]
Monthly expenses with metastatic tumor (1st year)	USD 2086.42	**γ**	Hollenbeak et al., 2011 [30]
Monthly expenses with metastatic tumor (after 1st year)	USD 810.58	**γ**	Hollenbeak et al., 2011 [30]
**Utilities**			
QoL of patients without tumor	1	**β**	Assumption
QoL of patients with metastatic tumor	0.66	**β**	De Groot et al., 2018 [31]
QoL of patients with detected tumor	0.75	**β**	De Groot et al., 2018 [31]
Death	0		Assumption
**Transition possibility**			
Efficacy of initial non-R0 resection	5.73%	**β**	Orosco et al., 2018 [32]
Possibility of local recurrence after resection	10.75%	**β**	Bradshaw et al., 2020 [11]
Risk of metastases without present tumor	1.00%	**β**	Bensalah et al., 2008 [10]
Possibility of occurrence of metastases	13.00%	**β**	Bensalah et al., 2008 [10]
Possibility of successful surgery of local recurrence	41.20%	**β**	Thomas et al., 2015 [33]
Additional risk of death with metastases	35.00%	**β**	Noone et al., 2018 [34]
Additional risk of death with localized tumor	3.50%	**β**	Assumption
Risk of death without tumor	(Age dependent)	**β**	US Life Tables 2015

## Data Availability

Not applicable.

## Supplementary Materials

The following supporting information can be downloaded at: https://www.mdpi.com/article/10.3390/cancers14092235/s1, Figure S1: CHEERS Checklist.
